# Anti‐saccade can be used as a screening tool for early cognitive impairment: a correlation study based on anti‐saccade parameters and cognitive function

**DOI:** 10.1111/psyg.13215

**Published:** 2024-11-08

**Authors:** Liwen Yang, Lingmei Lu

**Affiliations:** ^1^ Department of Neurology Qidong People's Hospital / Affiliated Qidong Hospital of Nantong University China

**Keywords:** anti‐saccade, cognitive impairment, error rates, reaction time

## Abstract

**Background:**

Eye movement tasks, especially anti‐saccade tasks, have been used to assess cognitive function in patients with neuropsychiatric disorders. Although it has been shown that individuals with cognitive impairment perform worse on anti‐saccades tasks, there is a lack of systematic evaluation of the sensitivity of parameters of anti‐saccades to assess different subtypes of cognitive impairment.

**Methods:**

A total of 158 participants were enrolled in this study, consisting of 66 men and 92 women, with an average age of 50.2 ± 10 years. The comparison of pro‐saccade reaction time, anti‐saccade reaction time, and error rates in the saccade task between individuals with cognitive impairments and a normal group was conducted. Furthermore, we systematically analyzed the correlations between the performance in neurological function tests (Mini‐Mental State Examination (MMSE), Montreal Cognitive Assessment (MoCA), Stroop) and these anti‐saccade parameters. Especially, the correlation between these parameters and cognitive function in different domains of the MoCA task were also evaluated.

**Results:**

The pro‐saccade reaction time, anti‐saccade reaction time and error rate were negatively correlated with the MMSE and MoCA scores (*P* < 0.001), and positively correlated with the time used in Stroop tasks. Among them, the error rate had the strongest correlation with the performance of MMSE, MoCA and Stroop tasks (MoCA: *P* < 0.0001, *r* ^2^ = −0.608; MMSE: *P* < 0.0001, *r* ^2^ = −0.344; Stroop: *P* < 0.0001, *r* ^2^ = 0.455). Among the seven cognitive domains examined by the MoCA task, error rates had relatively high correlations with visuospatial/executive (*P* < 0.0001, *r* ^2^ = −0.4660) and delayed recall (*P* < 0.0001, *r* ^2^ = −0.4228) compared to naming, language (*P* = 0.0004, *r* ^2^ = −0.0788), attention (*P* = 0.0004, *r* ^2^ = −0.0780), abstraction (*P* < 0.0001, *r* ^2^ = −0.1515), orientation (*P* < 0.0001, *r* ^2^ = −0.1075). Moreover, pro‐saccade reaction time, anti‐saccade reaction time and error rate of people with high MoCA scores were significantly higher than those of people with low MoCA scores, which can be used to identify people with mild cognitive impairment.

**Conclusions:**

Our study's results provide valuable clinical evidence supporting the effectiveness of anti‐saccades in assessing cognitive impairment, which is beneficial for screening and timely clinical intervention in individuals with specific cognitive impairment.

## INTRODUCTION

Dementia globally ranks among the primary causes of disability in elderly individuals.^1^ As the disease advances, it gradually impairs the patient's memory, cognitive abilities, and social skills, significantly impacting their daily life and imposing substantial economic and psychological burdens on both the family and society.^1^ However, an effective treatment strategy is still lacking.^2^ Hence, the accurate diagnosis and timely intervention of mild cognitive impairment (MCI), an early stage of dementia, are deemed crucial in preventing its onset.

Currently, the most utilised assessments for evaluating cognitive function in the diagnosis of MCI are neuropsychological scales, including the Mini‐Mental State Examination (MMSE) and the Montreal Cognitive Assessment (MoCA).^3^, ^4^, ^5^ However, the administration of these tests necessitates trained medical professionals or general practitioners, resulting in substantial labour expenses. Additionally, these psychological evaluations are relatively time‐consuming. For instance, the MMSE typically requires 20 min to complete, with the duration potentially extending for older or less educated individuals.^6^ Consequently, there is a pressing need to identify more expedient and user‐friendly methods for MCI assessment.

Saccades refer to rapid and jerky movements of the eyes. In the anti‐saccade task, an object is intentionally presented on one side of the screen, and participants are instructed to redirect their gaze to the opposite side.^7^ During this process, parameters such as reaction time and error rates are measured and recorded.^8^ The performance of the anti‐saccade task is influenced by several cognitive functions, including attention, target selection, memory, decision‐making, and intention.^9^ The control of saccades relies on the coordinated activity of multiple brain areas, including the cortex and subcortex.^10^ Impairment in any of these brain regions can impede the completion of specific tasks. Consequently, the anti‐saccade task is a valuable tool to assess cognitive function with the advantages of non‐invasiveness and convenience.^11^ However, previous studies have predominantly focused on saccadic disorders resulting from specific neurological injuries or diseases, with limited research systematically evaluating the relationship between various parameters in anti‐saccadic tasks and neuropsychological test results.

In this study, we conducted a comparison of the correlations between various parameters, including pro‐saccade reaction time, anti‐saccade reaction time, and anti‐saccade error rate, with MMSE, MoCA, and Stroop performance. Furthermore, we examined the disparities in anti‐saccade parameters between individuals with high and low MoCA scores. Additionally, we systematically evaluated the sensitivity and specificity of these parameters in predicting cognitive impairment. The aim of this research was to offer data reference for the utilisation of the anti‐saccade task in the screening of individuals with MCI.

## MATERIALS AND METHODS

### Participants

The participants in this study were recruited from the Neurology Department outpatient clinic at Qidong People's Hospital. The inclusion criteria were as follows:

1. age range: individuals aged 40 to 60 years
2. neurological status: patients diagnosed with neurological conditions relevant to the study focus but without other comorbid neurological or psychiatric disorders that could influence cognitive function
3. cognitive function: all participants had to be capable of completing cognitive function tests, psychological assessments, and activities of daily living without external assistance.

The exclusion criteria were refined as follows:

1. neurological and psychiatric disorders: patients with other neurological or psychiatric conditions (e.g., Alzheimer's disease, schizophrenia, epilepsy) that could affect cognitive performance or influence the results of the administered tests (MoCA, MMSE, Stroop test, and anti‐saccade task) were excluded
2. auditory and visual function: participants with severe auditory or visual impairments, including significant hearing loss or uncorrected visual impairment, that could hinder performance on the cognitive function tests were excluded
3. ocular health: any patient with ocular conditions (e.g., cataracts, glaucoma, severe dry eye, retinal disorders) or structural eye abnormalities that could interfere with the performance or interpretation of the anti‐saccade task was excluded
4. general health: patients with severe systemic diseases (e.g., advanced cardiovascular disease, uncontrolled diabetes) or any other medical condition that could impact cognitive or visual performance were also excluded.

A neurologist administered assessments of the MoCA, MMSE, Stroop test, and anti‐saccade test to all patients, following standardised procedures. Only participants with complete data on socio‐demographic characteristics, medical history, cognitive function tests, psychological assessments, and activities of daily living were included in the study. This study received approval from the Ethics Committee of Qidong People's Hospital (ER‐XXM‐LWTG‐2022‐002), and informed consent was obtained from each participant.

### MoCA, MMSE and Stroop task

Both the MoCA and MMSE assessments were conducted face to face by a neurologist, following the guidelines and protocols, within a time frame of 5–10 min and 10–15 min respectively. The MoCA is designed to assess multiple cognitive domains that are often affected in MCI, including visuospatial/executive skills, naming, attention, language, abstraction, and delayed recall. Its comprehensive nature makes it sensitive to detecting subtle cognitive changes that may indicate MCI. The test involves various tasks, such as drawing a clock, recalling words, and performing simple calculations, which together provide a holistic view of an individual's cognitive abilities. A MoCA score of 26 or below is indicative of cognitive impairment, reflecting significant difficulties in these critical domains. It consists of a 30‐point scale. The MMSE primarily evaluates cognitive function across various domains, including orientation, attention, memory, and language, but it is more focused on basic cognitive tasks. Although it is widely used in clinical settings, its sensitivity in detecting MCI is lower compared to the MoCA. The MMSE involves straightforward tasks, such as naming objects, repeating phrases, and performing simple arithmetic.^12^ In MoCA tasks, MCI: MoCA score 19–25; normal: MoCA score >25. In the MMSE tasks: MCI: MMSE score 24–26; normal: MMSE score>26. After reevaluating the subjects included in this study, it was found that their MoCA scores were ≥19 and their MMSE scores were ≥24. The Stroop task was performed as previously described.^13^ The demographic information of the study participants is presented in Table 1.

**Table 1:** Demographic information

Participants (M/F)	158 (66/92)
Age, mean ± SD	50.2 ± 10
MoCA positive score	53 (23.4 ± 1.5)
MoCA negative score	105 (27.7 ± 1.3)
MMSE positive score	6 (26 ± 0)
MMSE negative score	152 (28.6 ± 1.1)

*Note*: MoCA positive (MCI): 19–25; MoCA negative (normal): score >25; MMSE positive (MCI): 24–26; MMSE negative: score >26. M, male; F, female; SD, standard deviation; MMSE, Mini‐Mental State Examination; MoCA, Montreal Cognitive Assessment.

### Pro‐saccade and anti‐saccade tasks

**Figure 1:**
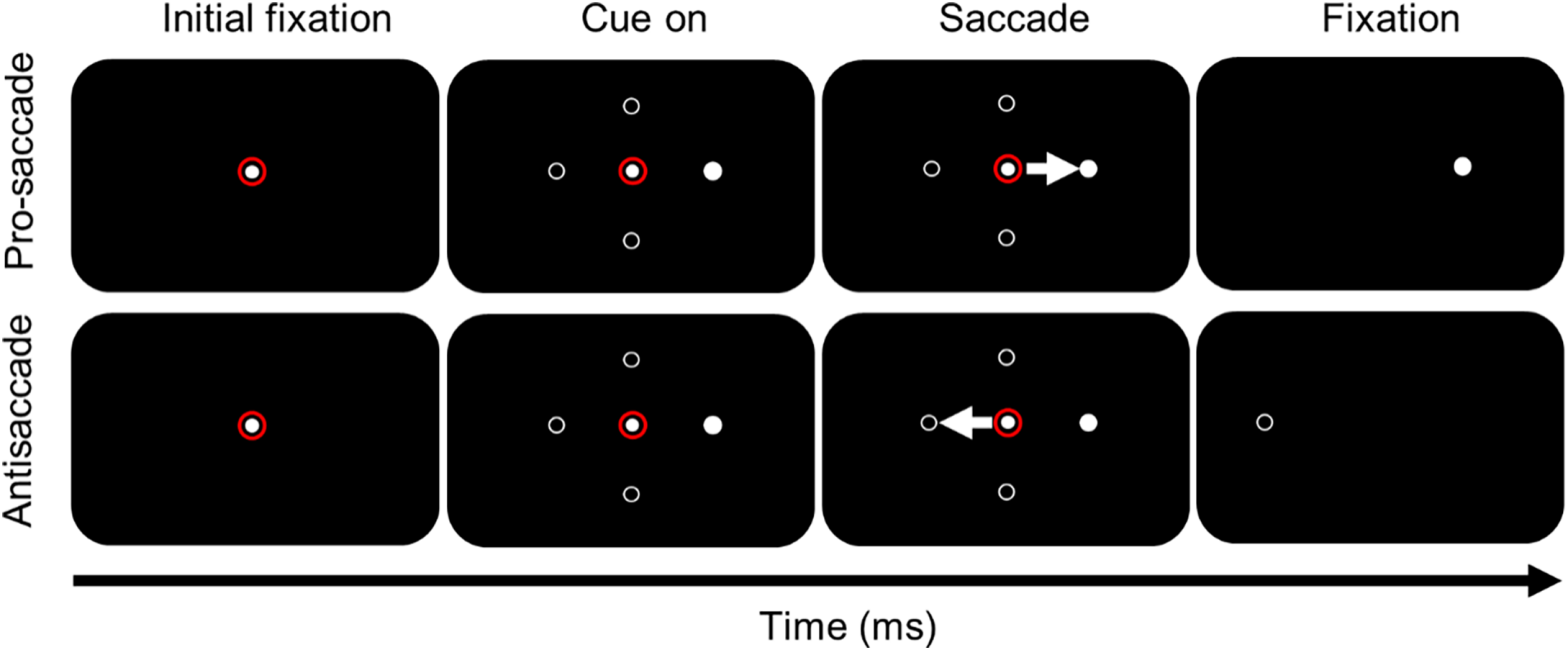
Schematic diagram of the saccade task. The red circle represents the required fixation point. The filled white circle represents the cue. The white hollow circle represents the potential cue locations. The white arrow was the saccade trajectory.

At the onset of the trial, participants were positioned 70 cm away from the display and their head was immobilised using a chin/forehead rest. Eye movements were tracked using VertiGoggles (ZT‐VNG‐II, Shanghai ZEHNIT Medical Technology Co., Ltd., Shanghai, China). During the pro‐saccade task, the initial target would appear on the left side of the horizontal display in the predictive saccade trial. Participants were required to promptly shift their gaze to the location where the fixation point emerged. The fixation point would stay on the screen for 800 ms before disappearing, followed by the appearance of a second target on the right side of the horizontal display. The left and right targets would alternate at predetermined positions and frequencies. Participants were instructed to execute saccades toward the visual cues with both accuracy and speed. Once the eye entered and remained within the examination window for 800 ms, the visual cue would vanish. The trial would conclude if no saccade was initiated within 2000 ms of the visual cue (Fig. 1).

In anti‐saccade trials, a target is initially presented at the centre of the display, and the duration of the display is randomised. Once the central target disappears, a first peripheral target is displayed at a random orientation (either horizontally to the left or right) and at a random angle (ranging from 10° to 20°). Following the disappearance of the first peripheral target, a second peripheral target immediately appears in the mirror position of the first. Participants are instructed to maintain fixation on the central target until any peripheral target appears. Then, they are required to make an anti‐saccade toward the mirror position of the first peripheral target as soon as it appears, and to continue looking at that location until the second peripheral target disappears (refer to Fig. 1).

### Data analysis

An unpaired *t*‐test was utilised to compare the differences in saccade parameters between subjects with higher and lower MoCA scores. Spearman rank correlation was employed to analyze the relationship between the saccade parameters of the anti‐saccade task and MMSE and MoCA scores. The *P*‐values and correlation coefficients (*r* ^2^) obtained from the correlation analysis indicate the significance of the regression fit and the strength of the correlation, respectively. The diagnostic efficacy of each saccade parameter in distinguishing patients with cognitive impairment from healthy individuals was assessed using the receiver operating characteristic (ROC) curve. The area under the ROC curve was used to evaluate the diagnostic efficiency. All statistical analyses in this study were performed using GraphPad Prism 8.0 software.

## RESULTS

### Pro‐saccade reaction time, anti‐saccade reaction time and error rate were correlated with MMSE, MoCA and Stroop performance

**Figure 2:**
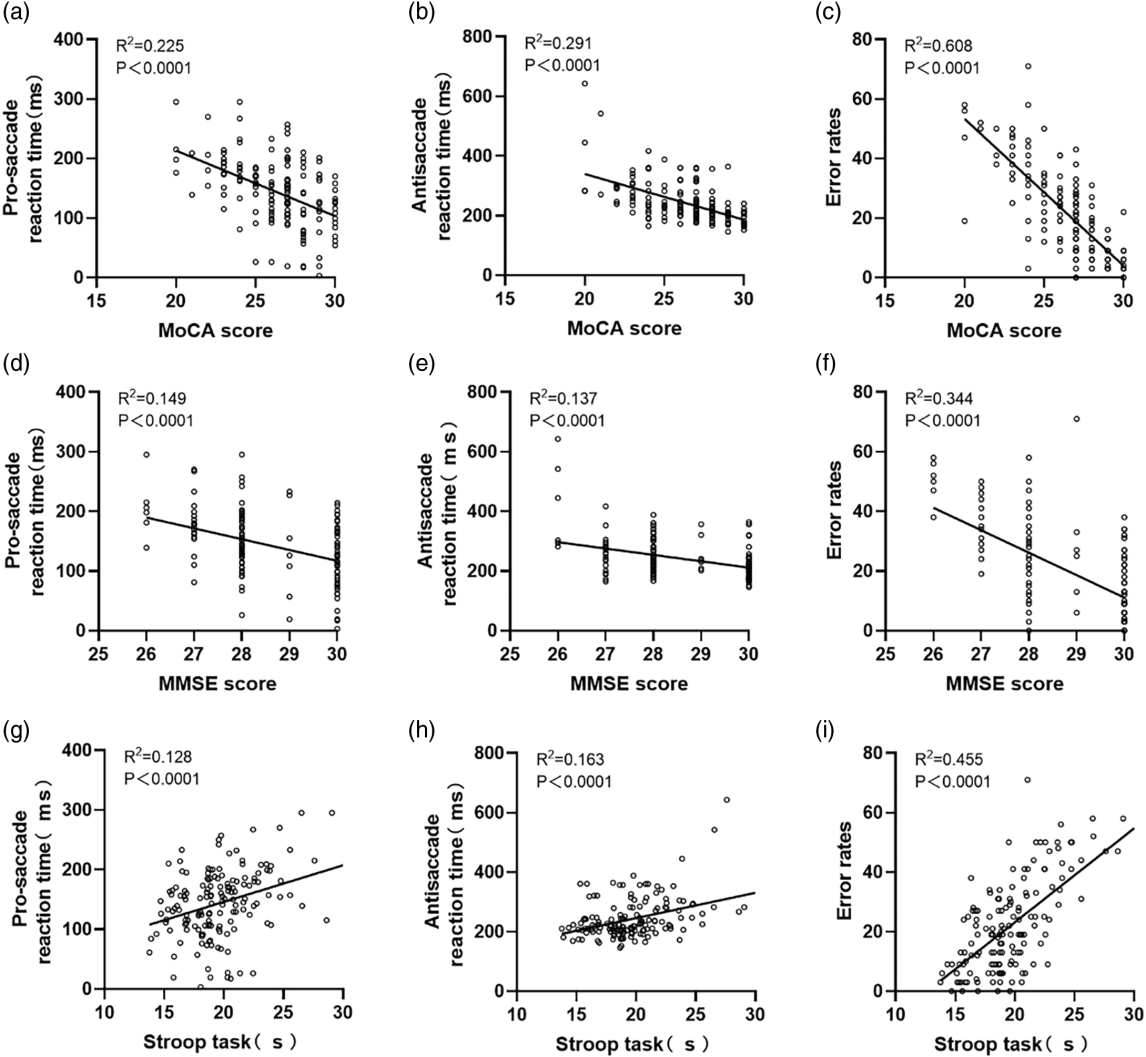
The correlation between each saccade parameter and Mini‐Mental State Examination (MMSE), Montreal Cognitive Assessment (MoCA) and Stroop test results was analyzed. Each hollow circle represents the test result for a single subject. (a–c) Pro‐saccade reaction time, anti‐saccade reaction time, correlation analysis between error rates and MoCA scores. (d–f) Pro‐saccade reaction time, anti‐saccade reaction time, (d–f) pro‐saccade reaction time. (g–i) Pro‐saccade reaction time, anti‐saccade reaction time, error rates and Stroop test time. The values of *P*, *R* ^2^ indicate the significance of the regression fit and the correlation coefficient.

Initially, we examined the correlation between three saccade parameters: pro‐saccade reaction time, anti‐saccade reaction time, and error rate, and their corresponding scores in the MoCA and MMSE tests for all included subjects. These parameters reflect the subject's ability, efficiency, and accuracy in performing the task. As shown in Fig. 2, the three parameters exhibited negative correlations with MMSE and MoCA scores, and positive correlations with the time spent to complete the Stroop task. It is noteworthy that compared to the pro‐saccade reaction time (MoCA: *P* < 0.0001, *r* ^2^ = −0.225, Fig. 2A; MMSE: *P* < 0.0001, *r* ^2^ = −0.149, Fig. 2D,G), the anti‐saccade reaction time (MoCA: *P* < 0.0001, *r* ^2^ = −0.291, Fig. 2B; MMSE: *P* < 0.0001, *r* ^2^ = −0.137, Fig. 2E; Stroop: *P* < 0.0001, *r* ^2^ = 0.163, Fig. 2H) exhibited weaker correlations with MoCA, MMSE, and Stroop performance, while the error rate (MoCA: *P* < 0.0001, *r* ^2^ = −0.608, Fig. 2C; MMSE: *P* < 0.0001, *r* ^2^ = −0.344, Fig. 2F; Stroop: *P* < 0.0001, *r* ^2^ = 0.455, Fig. 2I) demonstrated stronger correlations. The results indicated that while pro‐saccade reaction time, anti‐saccade reaction time, and error rate could partially explain the performance of subjects in MoCA, MMSE, and Stroop tests, it was the error rate that had the most significant impact on evaluating cognitive impairment.

### The correlation between anti‐saccade error rate and different functional domains in MoCA

**Figure 3:**
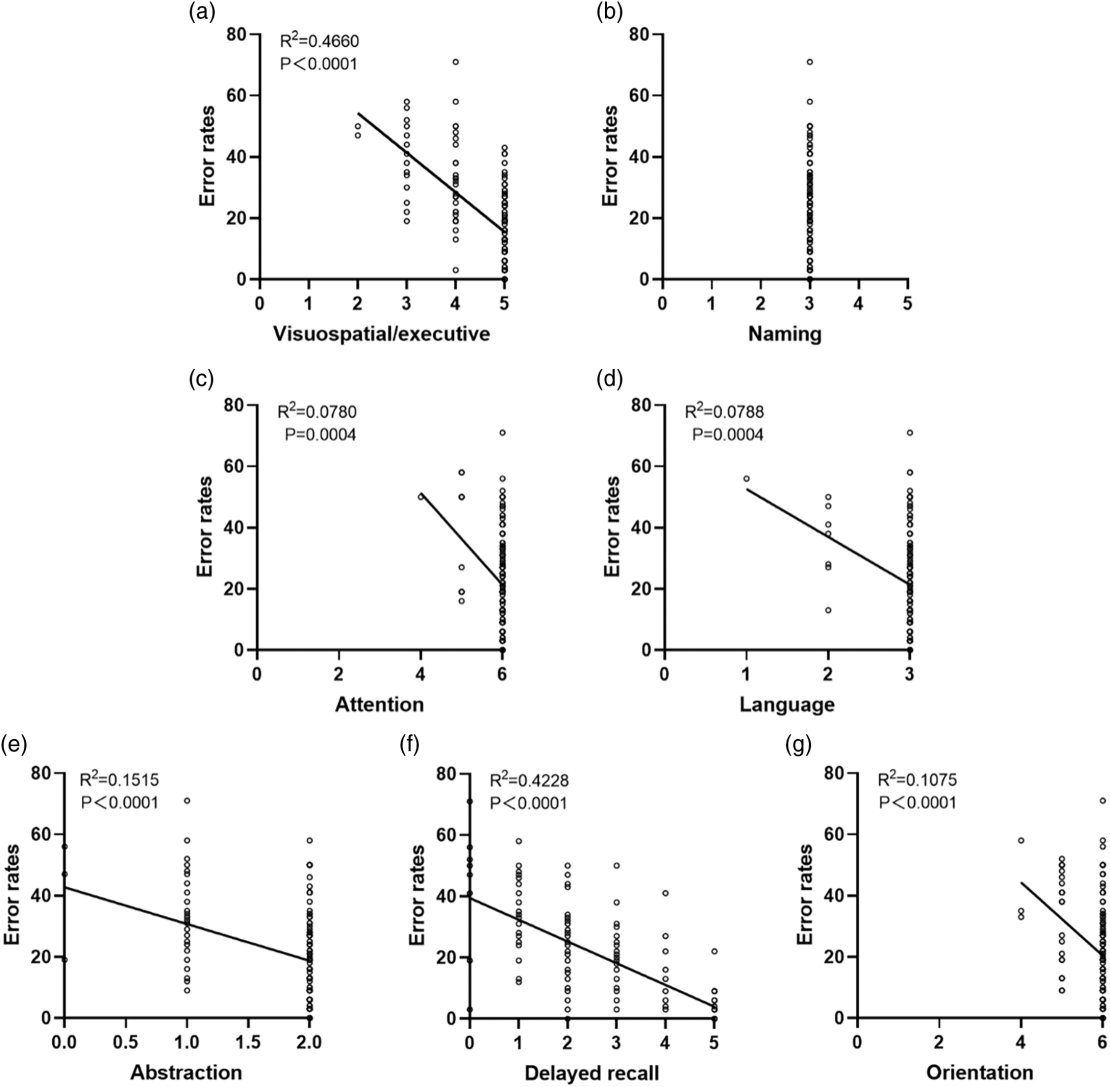
Correlation analysis between ability scores in seven domains of Montreal Cognitive Assessment (MoCA) and error rate (ER). (a) Correlation analysis between visuospatial/executive score and ER; (b) correlation analysis between naming score and ER; (c) correlation analysis between attention score and ER; (d) correlation analysis between language score and ER; (e) correlation analysis between abstraction and ER; (f) correlation analysis between delayed recall and ER; (g) correlation analysis between orientation and ER.

The findings above suggest that error rate may have a stronger correlation with MoCA scores compared to pro‐saccade reaction time and anti‐saccade reaction time. Consequently, we further examined the scores of seven domains in the MoCA test in relation to the error rate. These domains included visuospatial/executive, naming, attention, language, abstraction, delayed recall, and orientation. The results revealed no correlation between the error rate and naming ability (Fig. 3B). However, a weak correlation was observed for language (*P* = 0.0004, *r* ^2^ = −0.0788, Fig. 3D), attention (*P* = 0.0004, *r* ^2^ = −0.0780, Fig. 3C), abstraction (*P* < 0.0001, *r* ^2^ = −0.1515, Fig. 3E), orientation (*P* < 0.0001, *r* ^2^ = −0.1075, Fig. 3G), and other abilities. In contrast, significant differences were found in visuospatial/executive (*P* < 0.0001, *r* ^2^ = −0.4660, Fig. 3A) and delayed recall (*P* < 0.0001, *r* ^2^ = −0.4228, Fig. 3F) abilities, indicating a relatively stronger correlation. These results highlight the variability in the correlation between the error rate and specific domains of cognitive abilities, with visuospatial/executive and delayed recall abilities showing a higher correlation.

### The anti‐saccade error rate could distinguish the cognitive impairment and healthy groups

**Figure 4:**
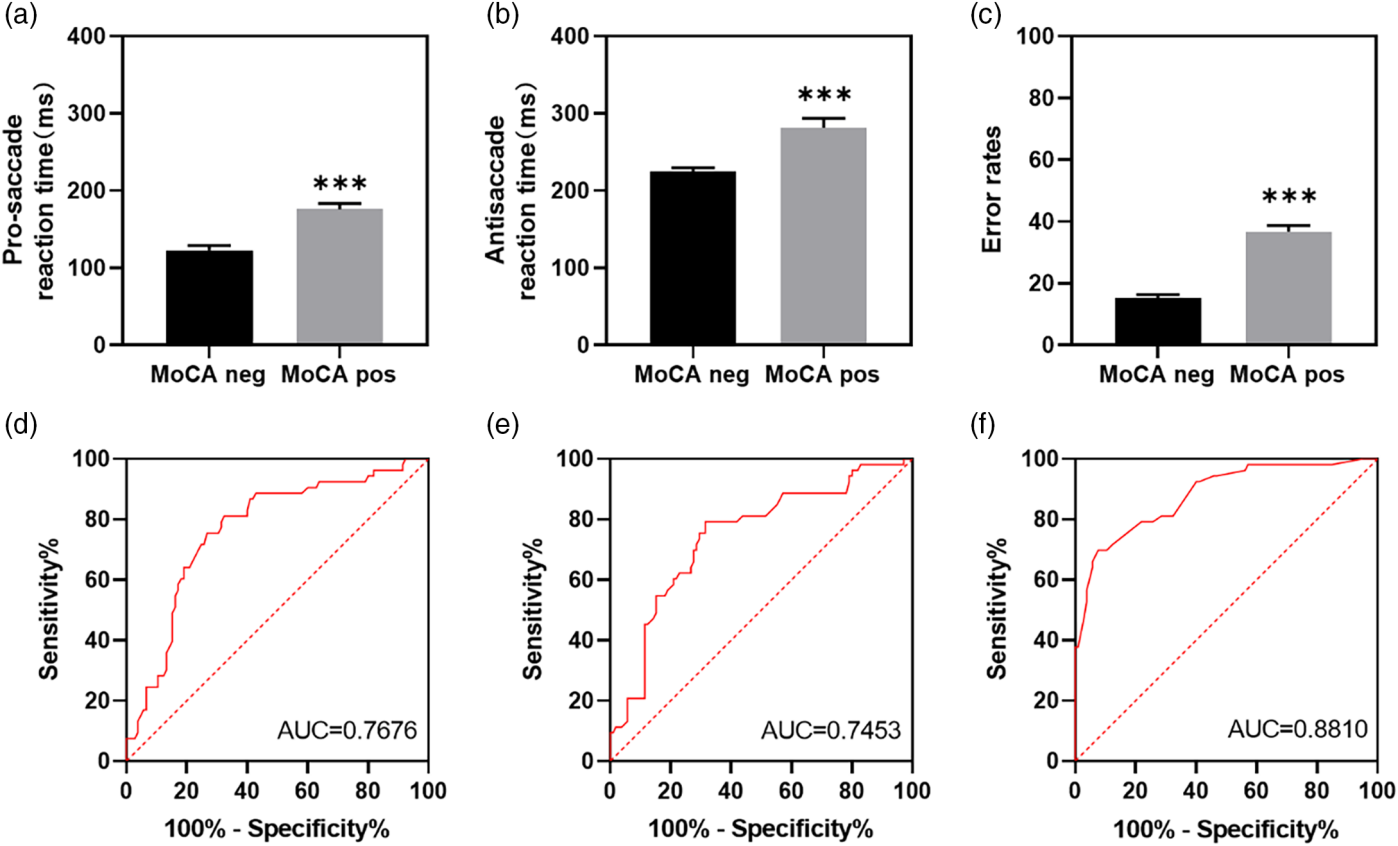
Differences and discrimination efficiency of different saccade parameters in cognitively impaired and normal subjects. (a‐c) Statistical analysis of pro‐saccade reaction time, anti‐saccade reaction time and error rates between cognitive impairment (Montreal Cognitive Assessment (MoCA) positive) and normal (MoCA negative) subjects; (d) Pro‐saccade reaction time receiver operating characteristic (ROC) curves based on MoCA positive and MoCA negative populations; (e) ROC curves of anti‐saccade reaction time based on MoCA positive and MoCA negative populations; (f) ROC plot of error rates based on MoCA positive and MoCA negative populations ***P < 0.0001 compared to MoCA negative group (unpaired *t*‐test).

Building upon these findings, we conducted a comparison of anti‐saccade parameters between individuals with cognitive impairment (MoCA positive) and normal healthy individuals (MoCA negative) on the MoCA test. The results demonstrated that the pro‐saccade reaction time in the MoCA positive group (Fig. 4A), as well as the anti‐saccade reaction time (Fig. 4B) and error rate (Fig. 4C), were significantly higher than those in the MoCA negative group. This indicates that these parameters hold potential for screening individuals with cognitive impairment. To assess individuals with cognitive impairment, we compared the accuracy of various saccade parameters. Specifically, we constructed a ROC curve based on the pro‐saccade reaction time of individuals who tested positive and negative on the MoCA. The results showed that the error rate (ER) had a higher area under the curve (AUC = 0.8810, 95% CI 0.8236–0.9383, Fig. 4F) compared to the pro‐saccade reaction time (AUC = 0.7676, 95% CI 0.6889–0.8463, Fig. 4D) and the anti‐saccade reaction time (AUC = 0.7453, 95% CI 0.6627–0.8279, Fig. 4E). Based on the ROC analysis, the optimal cut‐off point for the ER was determined using Youden's Index. At this threshold, the sensitivity was 69.81% and the specificity was 92.38%. For pro‐saccade reaction time, the optimal cut‐off point yielded a sensitivity of 71.7% and a specificity of 75.24%. For anti‐saccade reaction time, the sensitivity was 79.25% and the specificity was 68.57% at the optimal cut‐off point. Therefore, the ER remains the strongest predictor of MCI. These findings suggest that all three parameters have the potential to identify individuals with cognitive impairment, with the ER demonstrating the highest discrimination efficiency.

## DISCUSSION

MCI is a precursor to dementia, and early detection is crucial in delaying its progression.^14^ As there is currently no effective drug for dementia treatment, it is essential to identify cognitive impairment at the early stage. Eye movements are regulated by various brain regions associated with cognition, making them a potential preclinical method for detecting cognitive dysfunction.^15^, ^16^, ^17^ In this study, we compared the pro‐saccade reaction time, anti‐saccade reaction time, and ER in the anti‐saccade task among participants, along with their performance on the MoCA, MMSE and Stroop tests. Additionally, we examined the differences in these three parameters between individuals with normal cognition and those with cognitive impairment based on their MoCA scores. Our findings highlight the potential value of these three parameters, particularly the ER, in the initial diagnosis of MCI.

In the anti‐saccade task, participants are required to suppress the reflex response and instead generate a motor command to look in the opposite direction of the stimulus. Compared to visual guidance, executing an anti‐saccade task involves the normal functioning of multiple neural circuits, including the dorsolateral prefrontal cortex, supplementary eye field, frontal eye fields, anterior cingulate cortex, posterior parietal cortex, thalamus, and striatum.^7^, ^18^, ^19^, ^20^, ^21^ Consequently, anti‐saccade tasks activate a broader network compared to saccade tasks. Our findings demonstrate that the correlation between ER and cognitive function measured by MoCA, MMSE and Stroop task is stronger than that of pro‐saccade reactive time (Fig. 2), suggesting that anti‐saccade parameters may provide more comprehensive and valuable information for assessing MCI. A previous meta‐analysis of visual‐guided saccades revealed longer latencies in patients with attention deficit disorder, but not in patients with MCI, when compared to controls. However, our results showed that patients with MCI exhibited higher reaction times and error rates in both pro‐saccade and anti‐saccade tasks than normal subjects (Fig. 4A–C). This may be attributed to different subtypes of cognitive dysfunction among the enrolled participants. The role of reaction times in the differentiation of different subtypes of cognitive impairment needs to be further explored.

The anti‐saccade task involves a diverse range of cognitive processes, such as decision‐making, working memory, goal‐oriented behaviour, knowledge acquisition, and attention.^22^ Imaging studies provide evidence indicating that the fronto‐ocular area is of significant importance in the initiation of saccades and the execution of anti‐saccade tasks, as opposed to regular saccades.^23^, ^24^ Additionally, the superior and inferior precentral sulcus, para‐central sulcus, and intraparitic sulcus have been observed to be activated during both binocular and antiocular saccades.^25^ It is worth noting that the superior precentral sulcus exhibits greater activation during anti‐saccade trials in comparison to forward saccade trials.^25^ These findings demonstrate that specific functional brain areas are involved in these distinct neural functions. By thoroughly investigating the relationship between anti‐saccade parameters and different neural functions, valuable insights can be gained in identifying the targets of brain injuries. Building on this, we analyzed the correlation of ER with different functional domains involved in the MoCA task. We found that, compared to naming, attention, language, abstraction, orientation, and visuospatial/executive functions, delayed recall showed a stronger correlation with ER. These results suggest that ER may be a useful measure in evaluating brain regions related to visuospatial/executive (such as the left parahippocampal gyrus, frontoparietal circuitry, and cerebellar lobule VII)^26^, ^27^, ^28^ and delayed recall (including the hippocampus, posterior cingulate cortex/precuneus, inferior parietal lobule, and medial prefrontal cortex).^29^, ^30^

Previous studies have indicated that individuals with MCI exhibit higher anti‐saccade error rates compared to healthy individuals, underscoring the potential of this method for early detection of cognitive deficits.^31^, ^32^ To further validate our findings, we conducted additional analyses exploring the relationship between anti‐saccade error rates and performance on cognitive tests. Our results demonstrated significant correlations, highlighting the utility of anti‐saccade parameters in clinical assessments and their potential to identify individuals at risk for cognitive decline. This emphasises the importance of incorporating anti‐saccade tasks into comprehensive cognitive evaluations, particularly for early intervention strategies targeting individuals showing signs of cognitive impairment.

In this study, we observed that the correlation between ER and certain MoCA subdomains, such as attention and language, was weaker compared to other domains like visuospatial/executive function and delayed recall. This may be because anti‐saccade tasks are more directly related to visuospatial and executive functions, which are crucial for successfully completing these tasks. In contrast, attention and language abilities may be less directly engaged in the anti‐saccade task, which could explain the weaker correlations observed in these areas. We also acknowledge the potential impact of outliers on the correlation analysis, particularly in the attention and language domains. Therefore, future studies with larger sample sizes may help to further clarify these relationships and minimise the influence of outliers, providing stronger evidence for the cognitive domains most closely associated with anti‐saccade performance.

Although this study provides valuable insights into the potential utility of anti‐saccade parameters in identifying MCI, some limitations should be acknowledged. First, while the sample size was sufficient for an initial investigation, it may limit the generalisability of the findings. Larger and more diverse cohorts are needed to validate our conclusions across different populations and subtypes of cognitive impairment. Second, although our study demonstrates significant correlations between anti‐saccade task parameters and cognitive test performance, it is important to note that the cross‐sectional design precludes the establishment of causal relationships. The observed associations suggest that anti‐saccade performance is related to cognitive impairment, but we cannot infer whether one directly causes the other. Future longitudinal studies or experimental designs would be necessary to explore the potential causal links between anti‐saccade performance and cognitive decline.

One advantage of anti‐saccade tasks is their ability to objectively measure cognitive function with minimal language or education bias, unlike MoCA or MMSE, which can be influenced by literacy and cultural factors. However, we acknowledge that anti‐saccade testing may require specialised equipment and training, limiting its use in some clinical settings.

We propose using portable eye‐tracking devices to administer anti‐saccade tasks in outpatient settings, as these devices are becoming increasingly accessible and user‐friendly. These devices can rapidly collect data on saccadic reaction times and error rates, which can be used in conjunction with traditional cognitive assessments such as the MoCA or MMSE. Anti‐saccade tasks could be administered by trained technicians as part of routine neurocognitive evaluations for individuals at risk of MCI. The results could be analyzed in real‐time using automated algorithms, providing clinicians with immediate feedback on the patient's cognitive status. This approach would be especially valuable in primary care settings, where early detection of cognitive impairment is crucial for timely intervention.

## CONCLUSION

In summary, our findings suggest the potential of utilising anti‐saccade parameters as an initial screening tool for detecting subtle MCI, which aligns with the prevailing opinion. Furthermore, it is crucial to note that the validity of different parameters varies across different neurological function tests. Therefore, future studies should aim to investigate the specific role of each parameter in distinct neurological function domains. Additionally, establishing a multi‐indicator joint assessment model is necessary to enhance the sensitivity of cognitive impairment diagnosis and provide valuable insights into identifying specific brain areas affected by damage.

## FUNDING INFORMATION

This work was supported by the Program of Nantong Health Commission (MB2021096) and Grant of Nantong Municipal Medical Innovation Team.

## DISCLOSURE

The authors declared they have no conflicts of interest regarding this work.

## Data Availability

The experimental data used to support the findings of this study are available from the corresponding author upon request.
